# Supplementary material for: Modeling glioblastoma heterogeneity as a dynamic network of cell states
Source: Mol Syst Biol. 2021 Sep 16;17(9):e10105. doi: 10.15252/msb.202010105 (PMC8444284; doi:10.15252/msb.202010105)
Supplement: Supplementary file 6 — Source Data for Figure 5 [file MSB-17-e10105-s004.zip › Figure5A_sourcedata/GSEA_3071/nosigdb_stateI.GseaPreranked.1622795145664/NB_KI_CLUSTER17.html]

Details for gene set NB\_KI\_CLUSTER17[GSEA]

|  || Dataset | state13071 |
| Phenotype | NoPhenotypeAvailable |
| Upregulated in class | na\_pos |
| GeneSet | NB\_KI\_CLUSTER17 |
| Enrichment Score (ES) | 0.45075783 |
| Normalized Enrichment Score (NES) | 2.6507273 |
| Nominal p-value | 0.0 |
| FDR q-value | 0.0 |
| FWER p-Value | 0.0 |
Table: GSEA Results Summary

  

Fig 1: Enrichment plot: NB\_KI\_CLUSTER17      
 Profile of the Running ES Score & Positions of GeneSet Members on the Rank Ordered List

  

| PROBE | GENE SYMBOL | GENE\_TITLE | RANK IN GENE LIST | RANK METRIC SCORE | RUNNING ES | CORE ENRICHMENT || 1 | PCOLCE |  |  | 15 | 1.696 | 0.0231 | Yes |
| 2 | CFH |  |  | 29 | 1.523 | 0.0440 | Yes |
| 3 | MATN2 |  |  | 34 | 1.483 | 0.0669 | Yes |
| 4 | ITIH5 |  |  | 35 | 1.473 | 0.0908 | Yes |
| 5 | APOE |  |  | 60 | 1.294 | 0.1048 | Yes |
| 6 | PLXDC2 |  |  | 61 | 1.265 | 0.1253 | Yes |
| 7 | LRP1 |  |  | 84 | 1.160 | 0.1377 | Yes |
| 8 | PHLDA3 |  |  | 93 | 1.116 | 0.1534 | Yes |
| 9 | ITGAV |  |  | 117 | 1.029 | 0.1634 | Yes |
| 10 | SVIL |  |  | 120 | 1.014 | 0.1792 | Yes |
| 11 | EMP1 |  |  | 122 | 1.012 | 0.1954 | Yes |
| 12 | CTSB |  |  | 129 | 0.964 | 0.2093 | Yes |
| 13 | ITPRIPL2 |  |  | 130 | 0.962 | 0.2249 | Yes |
| 14 | GPX8 |  |  | 133 | 0.959 | 0.2398 | Yes |
| 15 | FBN1 |  |  | 138 | 0.944 | 0.2540 | Yes |
| 16 | NPC2 |  |  | 139 | 0.943 | 0.2693 | Yes |
| 17 | AHNAK |  |  | 163 | 0.887 | 0.2769 | Yes |
| 18 | LAMA4 |  |  | 176 | 0.863 | 0.2874 | Yes |
| 19 | ANXA4 |  |  | 190 | 0.847 | 0.2973 | Yes |
| 20 | SPON2 |  |  | 218 | 0.789 | 0.3022 | Yes |
| 21 | AEBP1 |  |  | 236 | 0.761 | 0.3096 | Yes |
| 22 | GBP1 |  |  | 248 | 0.736 | 0.3183 | Yes |
| 23 | DAB2 |  |  | 277 | 0.702 | 0.3215 | Yes |
| 24 | S100A13 |  |  | 306 | 0.665 | 0.3241 | Yes |
| 25 | GRN |  |  | 321 | 0.655 | 0.3307 | Yes |
| 26 | LRP10 |  |  | 329 | 0.646 | 0.3391 | Yes |
| 27 | LAMP2 |  |  | 352 | 0.629 | 0.3429 | Yes |
| 28 | PROCR |  |  | 355 | 0.627 | 0.3524 | Yes |
| 29 | RASSF4 |  |  | 384 | 0.603 | 0.3540 | Yes |
| 30 | ANXA5 |  |  | 392 | 0.597 | 0.3617 | Yes |
| 31 | TNS3 |  |  | 399 | 0.588 | 0.3695 | Yes |
| 32 | PLA2G16 |  |  | 408 | 0.578 | 0.3765 | Yes |
| 33 | FTH1 |  |  | 414 | 0.578 | 0.3844 | Yes |
| 34 | ARHGAP24 |  |  | 425 | 0.573 | 0.3908 | Yes |
| 35 | NOTCH2 |  |  | 446 | 0.551 | 0.3939 | Yes |
| 36 | CTSO |  |  | 472 | 0.532 | 0.3952 | Yes |
| 37 | CTSD |  |  | 474 | 0.531 | 0.4035 | Yes |
| 38 | TNS2 |  |  | 482 | 0.526 | 0.4100 | Yes |
| 39 | DNAJB4 |  |  | 503 | 0.507 | 0.4124 | Yes |
| 40 | BLVRB |  |  | 535 | 0.488 | 0.4112 | Yes |
| 41 | HNMT |  |  | 540 | 0.487 | 0.4179 | Yes |
| 42 | VMP1 |  |  | 545 | 0.483 | 0.4246 | Yes |
| 43 | LAPTM4A |  |  | 553 | 0.478 | 0.4303 | Yes |
| 44 | FTL |  |  | 564 | 0.473 | 0.4351 | Yes |
| 45 | LOXL2 |  |  | 589 | 0.459 | 0.4355 | Yes |
| 46 | RHOB |  |  | 609 | 0.447 | 0.4372 | Yes |
| 47 | WIPI1 |  |  | 620 | 0.443 | 0.4414 | Yes |
| 48 | LAMC1 |  |  | 640 | 0.439 | 0.4430 | Yes |
| 49 | EGR1 |  |  | 652 | 0.432 | 0.4468 | Yes |
| 50 | CREB3L2 |  |  | 699 | 0.412 | 0.4400 | Yes |
| 51 | SPATS2L |  |  | 710 | 0.408 | 0.4437 | Yes |
| 52 | CSTB |  |  | 717 | 0.405 | 0.4485 | Yes |
| 53 | KLF4 |  |  | 742 | 0.400 | 0.4480 | Yes |
| 54 | PON2 |  |  | 770 | 0.392 | 0.4464 | Yes |
| 55 | TRAM1 |  |  | 780 | 0.389 | 0.4501 | Yes |
| 56 | CLIC4 |  |  | 800 | 0.382 | 0.4508 | Yes |
| 57 | NECTIN2 |  |  | 845 | 0.366 | 0.4438 | No |
| 58 | REXO2 |  |  | 916 | 0.348 | 0.4290 | No |
| 59 | IFI6 |  |  | 923 | 0.344 | 0.4328 | No |
| 60 | IGFBP6 |  |  | 1048 | 0.318 | 0.4017 | No |
| 61 | CD81 |  |  | 1059 | 0.315 | 0.4039 | No |
| 62 | MYDGF |  |  | 1067 | 0.313 | 0.4069 | No |
| 63 | PMP22 |  |  | 1069 | 0.312 | 0.4117 | No |
| 64 | GPNMB |  |  | 1070 | 0.312 | 0.4168 | No |
| 65 | PHACTR2 |  |  | 1142 | 0.299 | 0.4009 | No |
| 66 | EPB41L2 |  |  | 1170 | 0.295 | 0.3978 | No |
| 67 | IL6ST |  |  | 1215 | 0.285 | 0.3895 | No |
| 68 | RCN1 |  |  | 1250 | 0.277 | 0.3841 | No |
| 69 | MIR99AHG |  |  | 1267 | 0.274 | 0.3838 | No |
| 70 | PLPP1 |  |  | 1395 | 0.252 | 0.3508 | No |
| 71 | TGFBR2 |  |  | 1410 | 0.251 | 0.3508 | No |
| 72 | ERRFI1 |  |  | 1640 | -0.273 | 0.2882 | No |
| 73 | EDNRB |  |  | 1754 | -0.287 | 0.2598 | No |
| 74 | KCTD12 |  |  | 1756 | -0.288 | 0.2642 | No |
| 75 | PMEPA1 |  |  | 2107 | -0.336 | 0.1673 | No |
| 76 | TCIM |  |  | 2270 | -0.366 | 0.1258 | No |
| 77 | TSPAN15 |  |  | 2479 | -0.407 | 0.0716 | No |
| 78 | PLAT |  |  | 2501 | -0.415 | 0.0722 | No |
| 79 | GNG11 |  |  | 2554 | -0.427 | 0.0639 | No |
| 80 | HEG1 |  |  | 2714 | -0.475 | 0.0251 | No |
| 81 | PLK2 |  |  | 2720 | -0.476 | 0.0314 | No |
| 82 | DAG1 |  |  | 2938 | -0.566 | -0.0229 | No |
| 83 | MT1E |  |  | 2982 | -0.591 | -0.0259 | No |
| 84 | ZEB2 |  |  | 3031 | -0.620 | -0.0299 | No |
| 85 | ERBB3 |  |  | 3038 | -0.625 | -0.0215 | No |
| 86 | NFIC |  |  | 3133 | -0.707 | -0.0376 | No |
| 87 | SPRY1 |  |  | 3163 | -0.746 | -0.0339 | No |
| 88 | IL1RAP |  |  | 3239 | -0.831 | -0.0424 | No |
| 89 | PLPP3 |  |  | 3335 | -0.988 | -0.0542 | No |
| 90 | EMP2 |  |  | 3338 | -0.998 | -0.0386 | No |
| 91 | NBL1 |  |  | 3432 | -1.368 | -0.0436 | No |
| 92 | GPM6B |  |  | 3478 | -1.861 | -0.0266 | No |
| 93 | NFIA |  |  | 3492 | -2.214 | 0.0056 | No |
Table: GSEA details [plain text format]

  

Fig 2: NB\_KI\_CLUSTER17: Random ES distribution      
 Gene set null distribution of ES for **NB\_KI\_CLUSTER17**
